# Namibia’s path toward malaria elimination: a case study of malaria strategies and costs along the northern border

**DOI:** 10.1186/1471-2458-14-1190

**Published:** 2014-11-20

**Authors:** Cara Smith Gueye, Michelle Gerigk, Gretchen Newby, Chris Lourenco, Petrina Uusiku, Jenny Liu

**Affiliations:** UCSF Global Health Group, San Francisco, CA USA; Clinton Health Access Initiative, Boston, MA USA; Namibia National Vector-borne Diseases Control Programme, Windhoek, Namibia; UCSF Global Health Sciences, 550 16th Street, 3rd Floor, UCSF Mail Stop 1224, San Francisco, CA 94158 USA

**Keywords:** Malaria, Malaria elimination, Namibia, Program operations, Costs

## Abstract

**Background:**

Low malaria transmission in Namibia suggests that elimination is possible, but the risk of imported malaria from Angola remains a challenge. This case study reviews the early transition of a program shift from malaria control to elimination in three northern regions of Namibia that comprise the Trans-Kunene Malaria Initiative (TKMI): Kunene, Omusati, and Ohangwena.

**Methods:**

Thirty-four key informant interviews were conducted and epidemiological and intervention data were assembled for 1995 to 2013. Malaria expenditure records were collected for each region for 2009, 2010, and 2011, representing the start of the transition from control to elimination. Interviews and expenditure data were analyzed across activity and expenditure type.

**Results:**

Incidence has declined in all regions since 2004; cases are concentrated in the border zone. Expenditures in the three study regions have declined, from an average of $6.10 per person at risk per year in 2009 to an average of $3.61 in 2011. The proportion of spending allocated for diagnosis and treatment declined while that for vector control increased. Indoor residual spraying is the main intervention, but coverage varies, related to acceptability, mobility, accessibility, insecticide stockouts and staff shortages. Bed net distribution was scaled up beginning in 2005, assisted by NGO partners in later years, but coverage was highly variable. Distribution of rapid diagnostic tests in 2005 resulted in more accurate diagnosis and can help explain the large decline in cases beginning in 2006; however, challenges in personnel training and supervision remained during the expenditure study period of 2009 to 2011.

**Conclusions:**

In addition to allocating sufficient human resources to vector control activities, developing a greater emphasis on surveillance will be central to the ongoing program shift from control to elimination, particularly in light of the malaria importation challenges experienced in the northern border regions. While overall program resources may continue on a downward trajectory, the program will be well positioned to actively eliminate the remaining foci of malaria if greater resources are allocated toward surveillance efforts.

## Background

While many countries in sub-Saharan Africa continue to scale-up malaria control measures [1], countries in Southern Africa are progressing toward elimination. Elimination is defined as the “reduction to zero of the incidence of infection caused by human malaria parasites in a defined geographical areas as a result of deliberate efforts” [1]. Since 2000, Namibia, South Africa, and Swaziland have all reduced malaria case incidence by more than 75%, and Botswana has relatively low malaria incidence as well [1]. However, pockets of transmission remain, primarily in northern border areas where malaria receptivity remains high and vulnerability is greater due to continuous population movement from neighboring endemic countries [2, 3]. Human migration from endemic to lower transmission areas can place destination countries at risk for malaria outbreaks or resurgence. Yet little is known about the types of program strategies and resource allocations required to reduce transmission in these vulnerable and highly porous border areas.

This case study aims to fill this evidence gap by examining the Namibia National Vector-borne Diseases Control Programme’s (NVDCP) strategies and activities during the early phases of its transition from malaria control to elimination, from 2000 to 2013. Malaria programs in three regions with moderate transmission that experience malaria importation from Angola—Kunene, Omusati, and Ohangwena—are described through archival record retrieval, literature review, and key informant interviews. Program implementation processes, intervention coverage, and epidemiological data are compared in order to identify the main technical, operational, and financial barriers encountered in regions with substantial cross-border challenges, and to highlight potential solutions. Along with broader implications for national malaria control programs in other countries on their way to eliminating malaria, insights for furthering Namibia’s malaria elimination strategy are discussed.

## Methods

This case study employed a mixed method approach, including historical record review, key informant interviews, and extraction of expenditure data from program accounts.

### Ethics statement

Approval for this study was obtained from institutional review boards of the University of California, San Francisco (12–09421) and the Namibia Ministry of Health and Social Services (P/Bag 13198).

### Sample selection

Three regions in Namibia—Kunene, Omusati, and Ohangwena—were purposefully chosen because of their relatively higher malaria transmission patterns and location bordering Angola. As each region is unique in its topography, climate and malaria epidemiology, the three regions together provide a range in setting for the programmatic and expenditure analysis. These regions are also a part of the Trans-Kunene Malaria Initiative (TKMI), a joint program between the Ministries of Health of Namibia and Angola. Expenditure data were collected for three consecutive years in each region: 2009, 2010, and 2011, representing the program’s early transition from malaria control to controlled low-endemic malaria.

### Data collection

From March to April 2013, researchers visited the three study regions and conducted thirty-four key informant interviews. Key informants were purposefully selected based on current or past experience in working with local malaria programs in the selected regions. Key informants also referred interviewers to other potential study participants at the conclusion of each interview. Potential study participants were either approached in-person if they were present in the health office or contacted through phone to set up meetings. Key informants included program directors, nurses, and environmental health assistants at different government levels, and representatives from private sector program collaborators. Interviews and data collection began at the national level, followed by visits to regional and district hospitals and health centers.

After obtaining informed verbal consent, interviews were conducted in English and audio-recorded. The interviewers followed a semi-structured questionnaire focused on program strategies, activities, history, epidemiological trends, and organizational structure. A second semi-structured questionnaire was used to elicit information about program expenditures and sources of financial records for program activities. At the end of each interview, key informants were asked to identify other individuals with knowledge of the covered topics.

Data on malaria epidemiology, malaria control intervention coverage, and demographics for 1995 to 2013 were collated from the NVDCP weekly surveillance system, Health Information System database, and NIP database. There were many gaps in epidemiological data, particularly for the number of indigenous and imported cases, as the surveillance system was not yet designed to capture this information. Population at risk (PAR) estimates and surveillance and vector control intervention coverage were also not available in many cases. Expenditure records were collected for all malaria activities for the years 2009, 2010, and 2011 from district, regional, and national offices. Only expenditures for the government-run program were captured, which included any external funding provided to the government (e.g., from GFATM grants) that was used for malaria control activities. Activities conducted by private sector organizations or NGOs and household out-of-pocket spending were not included. All available data sources were accessed and triangulated when possible. To account for differences in service delivery needs across regions, yearly expenditures were divided by the total population (the entire population of all three regions is classified as at risk by the NVDCP).

### Data analysis

Interview transcriptions were analyzed using a coding scheme developed to identify common themes, including risk groups, program strategies and interventions, financial and human resources, cross border activities, community involvement, challenges, and success factors. Expenditure data were analyzed across two dimensions:

1. malaria activity: diagnosis and treatment, prevention and vector control, surveillance, information and education campaigns, and program management and monitoring and evaluation (M/M&E); and
2. expenditure type: personnel, commodities, services, and capital equipment.

All expenditures were adjusted to 2011 prices and converted to US dollars. For additional details, see Appendix A. Information from interviews was then combined with expenditure data to understand the context in which malaria activities were carried out, enabling the identification of program strengths and constraints.

## Results

### Namibia’s malaria control efforts

More than 65% of Namibia’s population lives in the ten northern regions considered malaria endemic, where low or moderate malaria transmission occurs [4]. Across the country, the climate varies from arid and semi-arid to subtropical, with temperatures between 5°C and 40°C. Malaria occurs seasonally with periodic focal outbreaks, primarily influenced by rainfall patterns [5]. The main vector in Namibia is *Anopheles arabiensis*, which is common in areas with lower rainfall [6]. *Anopheles funestus* and *Anopheles gambiae* are also present, but have been greatly reduced in recent years [7]. Breeding areas for *An. arabiensis* are “iishanas”, or flat, low-lying areas that collect water during the rainy season and dry out during drought periods. *An. arabiensis* tends to feed at night, biting humans indoors as well as cattle outdoors [8]. This diversity in feeding behavior can make *An. arabiensis* more difficult to control using traditional vector control interventions. *Plasmodium falciparum* (*Pf*) accounts for 97% of all malaria cases [7].

Malaria in Namibia has recently undergone an epidemiologic transition [9]. Malaria control interventions have reduced endemic malaria transmission to a state of controlled low-endemic malaria (CLM), a level at which “malaria no longer constitutes a major public health burden, but at which transmission would continue to occur even in the absence of importation” [10]. Between 2001 and 2011, reported cases from health facilities declined from 562,703 to 14,406, and deaths attributed to malaria fell from 1,747 to 36—reductions of 97.4% and 98.0%, respectively. Substantial improvements in health and economic development also occurred during this period. Gross domestic product per capita has nearly tripled from US$1,830 in 2001 to US$5,380 in 2011, while life expectancy has increased from 57.3 to 62.3 years, and infant mortality has declined from 71.7 to 45.6 deaths per 1,000 live births [11].

**Figure 1:**
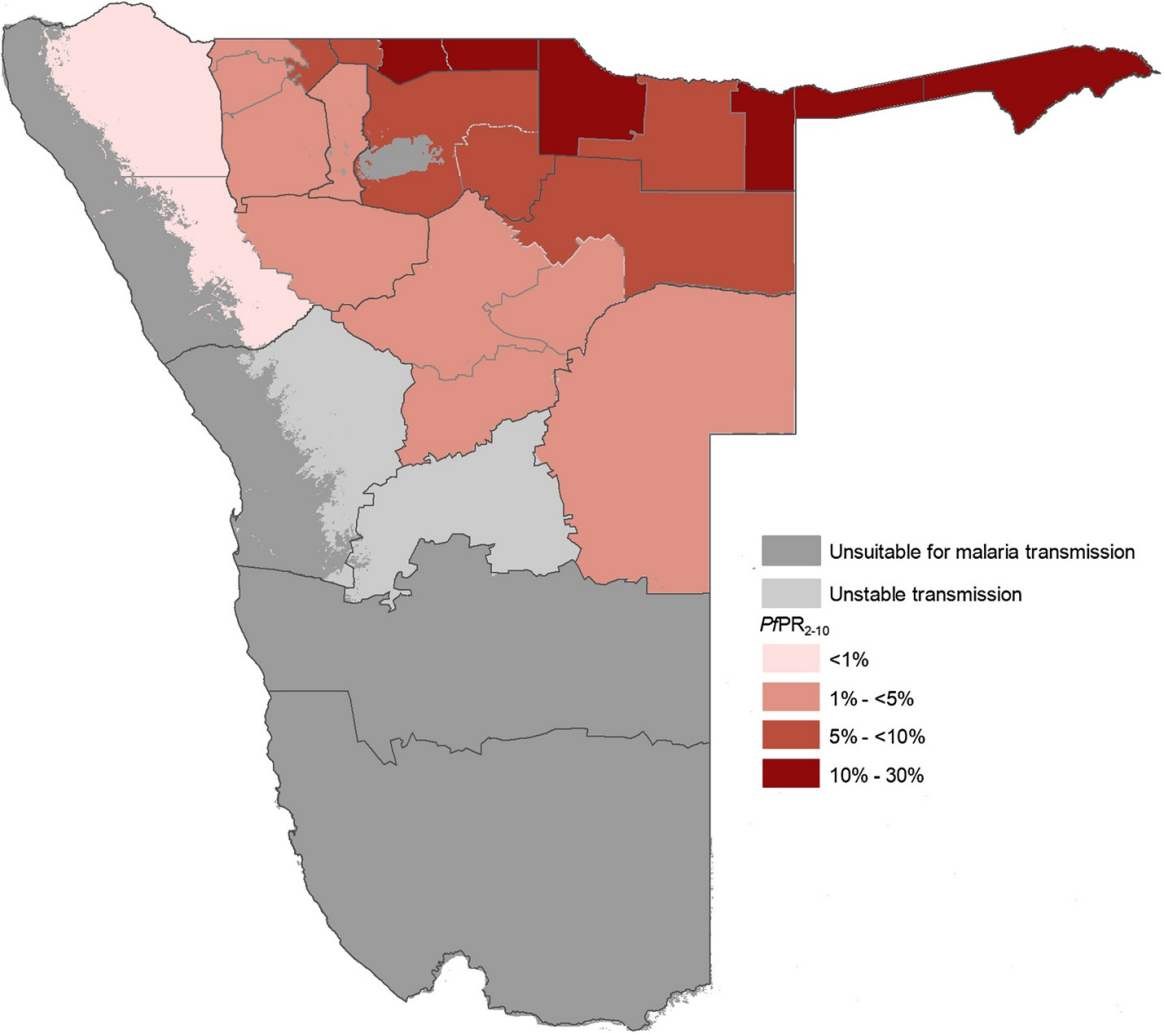
***P. falciparum*** **transmission and predictions of receptive** ***Pf*** **PR**_**2–10**_ **.** Map of Namibia showing the spatial limits of *P. falciparum* transmission and predictions of receptive *P. falciparum* parasite rate (for age range 2–10 years, or *Pf*PR_2–10_) at health district within the stable limits. The receptive risks were computed as the maximum mean population adjusted *Pf*PR_2–10_ predicted for the years 1969, 1974, 1979, 1984 and 1989 for each health district [13].

Despite the overall reduction of malaria, there remains low to moderate transmission in the northern regions bordering Angola [12]. Figure 1 describes the spatial limits of *Pf* transmission and predictions of receptivity. Of the three study regions, Ohangwena has the highest transmission receptivity potential, followed by Omusati and Kunene [13]. While the western coast of Kunene is unsuitable for malaria transmission, the northeastern area has stable controlled low-endemic transmission (*Pf*PR_2–10_ < 1%) and the southeast has hypoendemic 1 transmission (*Pf*PR_2-10_1 to <5%). Most of Omusati has hypoendemic 1 transmission, while the border area between Omusati and Ohangwena has hypoendemic 2 transmission (*Pf*PR_2–10_ 5 to <10%). The eastern parts of Ohangwena have mesoendemic transmission (*Pf*PR_2-10_10 to 30%). See Appendix A for methods used to generate Figure 1.

**Figure 2:**
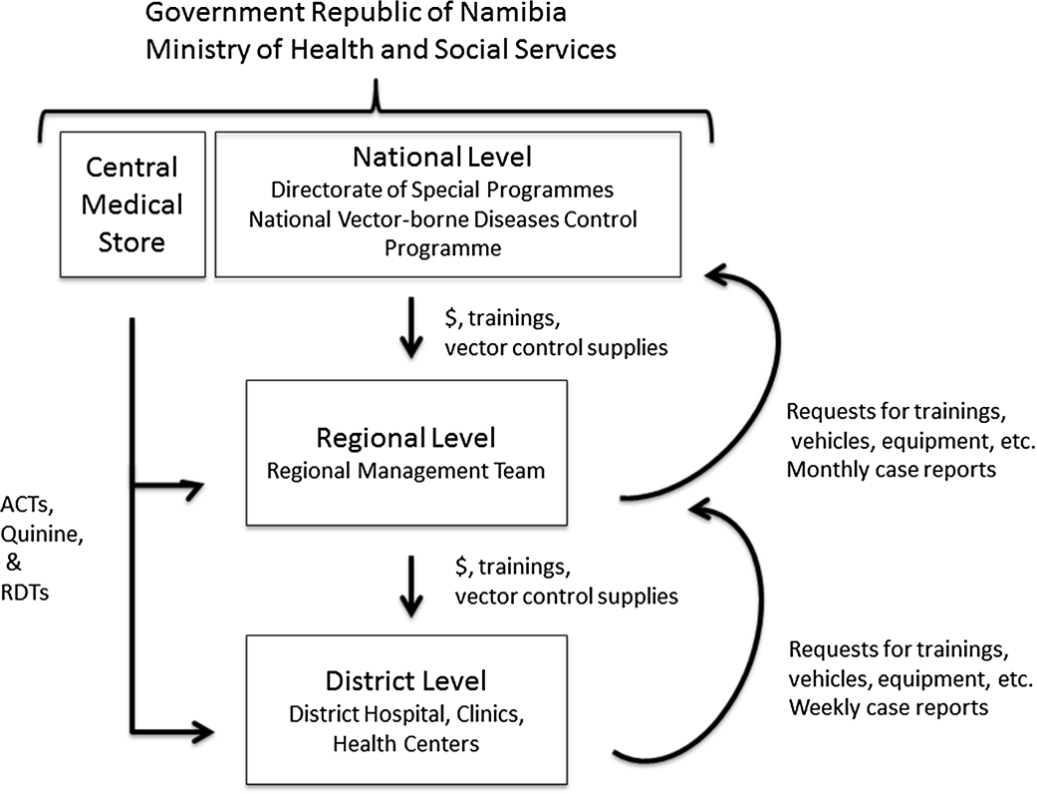
**Malaria program organization.** Within the Government Republic of Namibia Ministry of Health and Social Services, the National Vector-borne Diseases Control Programme is part of the Directorate of Special Programmes (DSP). At the national level, the program supervises malaria activities at the regional and district level, providing them with trainings and supplies for vector control. The Central Medical Store provides all medicines and clinical supplies required to carry out malaria case management. Regional DSP Programme Administrators and Environmental Health Officers organize and support activities at the regional and district levels.

Established in 1991, the NVDCP is based in both Windhoek, the capital of Namibia, and Oshakati, in the northern malaria endemic area. The Directorate of Special Programmes (DSP) is a directorate of the Ministry of Health and Social Services (MoHSS) that oversees all activities related to HIV/AIDS, tuberculosis, and vector-borne diseases, including malaria. Figure 2 depicts the organizational structure of the NVDCP. At the regional level, malaria services are managed by the Environmental Health Unit and DSP focal persons. At the district level, malaria activities (i.e. indoor residual spraying (IRS), diagnosis and treatment, and community outreach) are executed by the Primary Health Care supervisors and Environmental Health Officers (EHOs). At health centers and clinics, nurses provide case management services and distribute long-lasting insecticide-treated nets (LLINs). In some areas, non-governmental organizations (NGOs) help conduct information, education and communication (IEC) campaigns and distribute LLINs. All public health facilities receive clinical supplies from the Central Medical Store, which is housed separately under the Directorate of Tertiary Health Care and Clinical Support Services [14]. The National Institute of Pathology (NIP), which is state owned, conducts malaria microscopy in 37 laboratories throughout the country.

The NVDCP is financially supported by the government and the Global Fund to Fight AIDS, Tuberculosis and Malaria (GFATM). Since January 2005, Namibia has received $18.8 million USD in GFATM disbursements [15, 16] allocated to malaria programme activities. The current grant beginning July 2010 has been extended to June 2016 and will disburse an additional $7.3 million USD. In April 2010, the NVDCP launched a campaign to move the country to pre-elimination/elimination in the next five to 10 years [17] with a goal of reducing incidence to less than 1 per 1,000 total population in every district by 2016 and achieving national elimination, or zero local malaria cases, by 2020 [18].

### Kunene region

**Figure 3:**
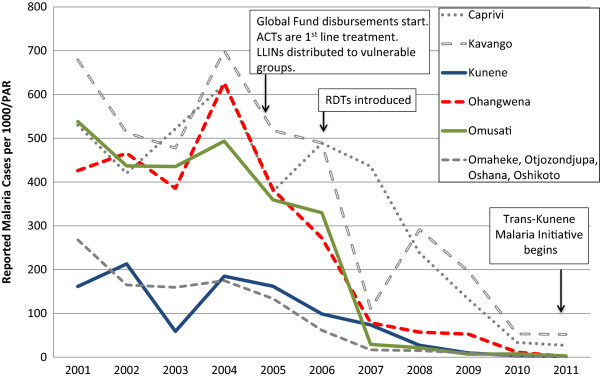
**Reported malaria cases from health facilities, 2001–2011.** Source: Health Information System, MoHSS Note: Region populations for 2002–2004 were not available. Calculated by taking difference between 2005 and 2001 populations, dividing by 4 and adding amount to each year. Note: Based on regional names and boundaries as of July 2013. The selected study regions are shown in color. Neighboring regions are shown for comparison. PAR = population at risk; ACT = artemisinin combination therapy; LLIN = long-lasting insecticide-treated nets; RDT = rapid diagnostic test.

Kunene is relatively remote and sparsely populated. Because the climate is mostly dry with only sporadic rainfall [19], the environment is not particularly receptive to mosquito breeding. However, vector larvae have been found in natural springs in the north near the Namibian-Angolan border, which is demarcated by the Kunene River and does not have any official border posts. Of three districts (Khorixas, Opuwo, and Outjo), Opuwo is the northernmost, the most populated, and has the highest malaria burden: 138 (88%) of the cases in 2011 in Kunene were reported from Opuwo. Kunene has fewer malaria cases than other northern regions, and the number of cases has declined, from 11,111 in 2001 to 729 in 2009 (API = 9.64) and further to 138 in 2011 (API = 1.52; see Figure 3, reported malaria cases).

**Figure 4:**
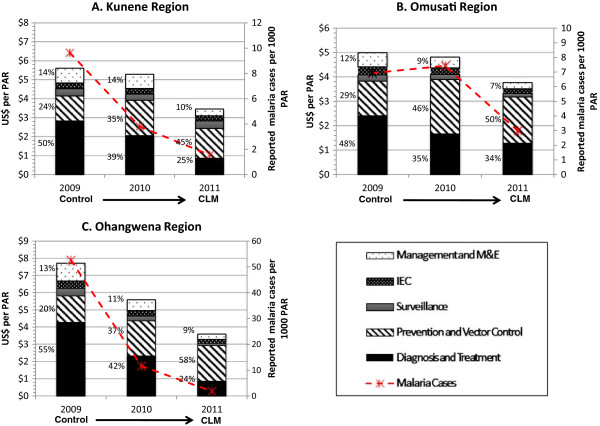
**Malaria program expenditures in study regions, 2009–2011.** PAR = population at risk; CLM = controlled low-endemic malaria; M&E = monitoring and evaluation. All figures are reported in 2011 USD. Note: Figures **A**, **B**, and **C** contain different scales in US$ per PAR.

From 2009 to 2011, total annual expenditures on malaria in Kunene declined by 28.0%, from US$ 5.61 per population at risk per year (PPY) to US$ 3.46 PPY in 2011 (see Figure 4, Panel A). Expenditures in the study include both government funding and the government funding provided from the GFATM grants. In 2009, diagnosis and treatment accounted for half of the total expenses (50.4%), followed by vector control and prevention (23.5%). By 2011, spending on diagnosis and treatment declined to 24.8%, most likely due to the decrease in treatment expenditures, but spending on vector control increased to 45.4%. Spending on personnel declined from 73.8% in 2009 to 66.5% in 2011, largely due to less time spent on diagnosis and treatment by health workers (see Table 1). Conversely, because of expanded IRS activity, spending on consumables increased from 9.3% to 20.6% over the same time period.

**Table 1:** **Population, malaria cases, and program expenditures for selected regions, 2009-2011**

	Control			Control/Controlled low-endemic malaria			Controlled low-endemic malaria		
	2009			2010			2011		
	Kunene	Ohangwena	Omusati	Kunene	Ohangwena	Omusati	Kunene	Ohangwena	Omusati
Total population	75,632	261,323	243,657	76,598	265,992	245,788	88,300	245,100	242,900
Reported^1^ malaria cases	729	13,755	1,689	292	3,078	1,828	138	451	729
Expenditures (US$2011)	$424,155	$2,015,576	$1,216,671	$405,194	$1,486,757	$1,183,450	$305,546	$881,399	$915,354
Expenditures/total population	$ 5.61	$ 7.56	$ 4.99	$ 5.29	$ 5.48	$ 4.81	$3.46	$4.02	$3.77
Expenditure type:									
Personnel	73.8% ($4.14)^2^	84.2% ($6.37)	73.6% ($3.67)	64.2% ($3.40)	71.7% ($3.93)	62.3% ($3.00)	66.5% ($2.30)	58.9% ($2.37)	50.8% ($1.92)
Consumables	9.3% ($0.52)	6.7% ($0.51)	18.1% ($0.90)	17.8% ($0.94)	18.8% ($1.03)	32.3% ($1.55)	20.6% ($0.71)	30.4% ($1.22)	47.2% ($1.77)
Services	15.8% ($0.89)	8.4% ($0.63)	7.1% ($0.36)	15.2% ($0.81)	7.5% ($0.41)	4.3% ($0.21)	7.2% ($0.25)	8.1% ($0.33)	0.9% ($0.03)
Capital	1.1% ($0.06)	0.7% ($0.05)	1.1% ($0.06)	2.7% ($0.14)	2.0% ($0.11)	1.1% ($0.05)	5.7% ($0.20)	2.6% ($0.10)	1.2% ($0.05)

^1^Reported from health facilities.

^2^Total cost per person at risk for particular expenditure type.

### Omusati region

Omusati, to the east of Kunene, is smaller in territory but more densely populated, particularly in the northern part of the region. Rainfall is more consistent in Omusati than Kunene [20]. Of four districts in Omusati, outpatient malaria cases in 2011 were highest in Outapi (130 cases), where an official border crossing exists, followed by Tsandi (113 cases), Oshikuku (35 cases), and Okahao (23 cases).

Malaria cases in Omusati declined from over 100,000 in 2001 to 5,256 in 2008 (see Figure 3). Between 2009 and 2011, cases dropped by 60.2%, from 1,689 (API = 6.93) to 729 (API = 2.77). Over the same time period, malaria program expenditures declined by 28.9%, from US$4.99 PPY to US$ 3.77 PPY, respectively (Figure 4, Panel B). Over this three-year period, the proportion of expenditures for diagnosis and treatment declined (from 48.2% to 34.0%) while the proportion for vector control and prevention increased (from 28.5% to 50.4%). These reductions were linked to a reduced proportional spending on personnel (from 73.6% to 50.8%), and increased proportional spending for consumables (from 18.1% to 47.2%; see Table 1).

### Ohangwena region

Of the three study regions, Ohangwena has the highest population density. While the area receives a considerable amount of precipitation relative to the other regions [21], rainfall is variable and droughts are common. Malaria cases declined from 97,338 in 2001 to 14,682 in 2008 (see Figure 3). From 2009 to 2011, total cases decreased by 77.6% (13,755 cases in 2009, API = 52.64; 451 cases in 2011, API = 1.69). Key informants believed that most cases originated in Angola: 87% of the region’s malaria cases were reported from Engela District, where the official border post is located.

From 2009 to 2011, malaria program expenditures dropped by 56.3% from US$ 7.71 PPY to US$ 3.60 PPY, the largest observed across all study regions (Figure 4, Panel C). Similar to the other regions, the proportion of spending over the three-year period declined for diagnosis and treatment (from 55.3% to 23.6%) and increased for vector control and prevention (from 20.0% to 57.6%). Expenditures for personnel also declined (from 82.4% to 58.9%) while expenditures for consumables increased (from 8.7% to 30.4%), mostly spent on insecticides (see Table 1).

### Cross-regional comparison of major malaria control interventions

A summary of the major technical, operational, and resource allocation challenges of the main malaria interventions elicited from key informants is provided in Table 2.

**Table 2:** **Technical, operational, and resource allocation challenges of key malaria interventions elicited from key informant interviews**

Issues discussed	Operational	Technical	Resource allocation
Indoor residual spraying	● Access and weather difficulties	● Does not cover mobile population	● Insufficient spray men
	● Homeowner/community refusals		● Delayed insecticide procurement in 2008
	● Late staff payments	● Not as effective against outdoor biting/resting vectors	
	● Turnover/retraining		● Irregular trainings
	● No documented strategy on targeting populations		● Lack of some IRS equipment
Long-lasting insecticide-treated nets	● Unclear/outdated targeting	● Does not cover mobile population	● Insufficient supplies of LLINs and resource mechanism for distribution
	● High turnover of community volunteers	● Not as effective against outdoor biting/resting vectors	
	● LLINs misused by recipients		● Insufficient IEC for proper use of LLINs
Diagnosis & treatment	● No official change in policy (until 2012)		● Trainings not organized or timed to coincide with new commodity rollout
	● No concentrated strategy across regions		
			● Insufficient IEC for dispelling myths and emphasizing need for prompt diagnosis and treatment
	● Some malaria patients reluctant to provide accurate contact or place of origin information.		
	● Some health workers perceive RDTs to be too time-consuming		
Surveillance/Reporting	● No analysis/feedback	● Reporting systems not linked across health system levels or regions	● Lack of personnel
	● Private sector not included		

### Indoor residual spraying

IRS, primarily with Dichloro-diphenyl-trichloroethane (DDT), has been the main malaria control intervention in Namibia since the 1960s [13]. Currently, DDT is mainly used on traditional structures (huts made of sticks and reeds) and deltamethrin is used on modern cement block structures. IRS is typically conducted from October to January, timed to start just before the onset of the rainy season, which lasts from November to April. IRS is coordinated and carried out by EHOs at regional and district levels who supervise spray teams comprised of temporary laborers, using insecticides and equipment provided by the national program. The national program also conducts supervisory visits during trainings and in the field, and conducts bioassay and susceptibility studies on the effectiveness of insecticides. Nationally, IRS coverage (i.e. percentage of the PAR that lives in an insecticide-treated structure) was 15.6% in 2008, and 48.9% of the population targeted for IRS were considered covered in that year. PAR is considered to be the total population in areas deemed at risk for malaria, which in the sampled regions includes the total population of the regions. In 2011, IRS coverage per PAR was 41.1% and the programme provided IRS coverage for 88.9% of the targeted population. The 2008 decline in coverage was caused by delayed procurement of insecticides.

In Kunene, 47.8% of the population was covered by IRS in 2011 (78.8% of population covered of those targeted). Spraying was concentrated in Opuwo District because it had more people and vector breeding sites. IRS coverage in Ohangwena was 38.4% (over 100% for population targeted), and in Omusati was 28.2% (93.1% of targeted). The insecticide shortage in 2008 caused IRS coverage in Ohangwena to decline to 5.0% of PAR (14.2% of population targeted). In Omusati, 5.4% of population at risk (28.3% of population targeted). Kunene was able to maintain coverage of 50.2% (over 100% of targeted population) reportedly because the region had leftover insecticide stocks from previous seasons which were used this year. Kunene has a smaller population density than Ohangwena and Omusati, and thus does not need as much insecticide.

In addition to occasional insecticide shortages, key informants noted that IRS training, while improved, still had some shortcomings. Prior to deployment each year, regional programs recruit teams of temporary spray men, who undergo a weeklong training that covers basic malaria information and IRS techniques. In recent years, this training has been expanded. For example, since 2011, a session on malaria case management has been included to increase community outreach and IEC by IRS teams, and to familiarize EHOs on reasoning behind newly introduced active case detection. In Ohangwena and Omusati, regional and district EHOs attributed increasing IRS coverage to better communication between the regional EHOs and community leaders. However, trainings are still only conducted when funding is available. For example, a 2009 training for regional officers covering basic entomology, malaria epidemiology, and planning did not happen again until 2013, with smaller-scale refresher trainings held each year in the interim.

Other operational constraints for IRS were related to community acceptability, access, and worker shortages. Key informants in Kunene described lower community acceptability related to fear of DDT exposure. IRS coverage for highly mobile pastoral populations is lower because they are often not at home when IRS teams arrive, and IRS would be less effective anyway as these individuals often sleep outside. In addition, community members are often unwilling to move belongings from their home to accommodate thorough spraying. In Kunene and Ohangwena, IRS progress has been hampered by poor roads, exacerbated by heavy rainfall.

Some of these operational challenges were reportedly linked to inadequate staffing. Spray activities could not be completed within four months because of the shortage of spray men. For example, EHO posts in Kunene were vacant for long periods. To avoid delays, the Ohangwena program recently attempted simultaneous IRS in different districts using smaller teams. Late payment of temporary spray men was an issue mentioned by key informants in all three regions, particularly in Omusati, and may have resulted in decreased morale and lower quality of IRS. The Omusati program also lacked equipment (e.g. tents) at times. In 2011, Omusati recruited 10 more spray men with GFATM funding to alleviate staffing shortages.

The timing of the spray season was another factor. IRS was planned to begin in October and end in January, overlapping with the rainy season. However, heavy rains and flooding made it difficult to reach certain areas, and older vehicles tended to break down in rough terrain. To avoid delays, the spray season was shifted in 2011 to start in September and end in December, but it has not yet been determined whether IRS coverage and quality have improved as a result.

### Long-lasting insecticide-treated nets

LLINs have been a main vector control method since the mid-2000s. Distribution of ITNs (targeting women only) began in 1993 in northern Namibia. A 2005 policy change instituted broader targeting of at-risk groups, including children under five years of age and pregnant women. From 2005 to 2011, over 625,000 LLINs were distributed at health facilities, outreach sites, antenatal clinics, and via mass campaigns to villages.

LLIN coverage (estimated at one net for two people for three years in at-risk populations targeted for LLINs by region, which are different across regions) varied across regions and years. Coverage in Kunene steadily increased from 6.1% in 2005 to 53.5% in 2009, but declined thereafter and was only 26.0% in 2011. Since 2005, coverage in Ohangwena increased from 9.0% to a peak of 43.9% in 2010, but declined to 30.5% in 2011. Similarly, coverage in Omusati increased from 10.3% (2005) to 52.8% (2010) before declining to 31.6% (2011). LLIN distribution was augmented in 2008 to compensate for lower IRS coverage.

In some regions, international and local NGOs helped to distribute LLINs and increase coverage. In Ohangwena, NGOs targeted entire villages and mobilized community volunteers to assist in delivery. This method appears to have been effective for mass distribution, but was hampered by high turnover of volunteers. Some communities refused to participate or use LLINs, even after meetings with local leaders. Starting in 2005, with support from GFATM, additional NGOs have distributed free and subsidized LLINs via social marketing [22]. Even though LLIN access has increased, challenges for further improving coverage remain. In Omusati, key informants reported insufficient supplies of LLINs for at-risk populations. In Kunene, because LLINs have been misused (e.g. draped on the outside of a structure), key informants stated that more education and involvement of traditional community leaders was needed.

In 2012, the NVDCP set a new goal to achieve 95% LLIN coverage of the entire population, shifting from just vulnerable populations to all those living in regions with any risk of malaria transmission by 2014 [15]. In 2013, a mass distribution of 87,900 LLINs was targeted to villages with the highest malaria caseloads in Zambezi, Kavango, and Omusati. By registering LLINs to each household, the program will be able to track recipients for future distributions and net replacement.

### Diagnosis/treatment: RDT and ACT rollout

Malaria diagnosis and treatment is available for free to both citizens and foreigners in all health facilities. Beginning in 2005, national guidelines called for clinical diagnosis with parasite confirmation using microscopy or a Rapid Diagnostic Test (RDT). RDTs were procured by GFATM and distributed for the first time in 2005, and were available in 90% of district health facilities by 2006. In 2011, a new RDT with improved sensitivity and specificity to *Pf* and the ability to test for multiple parasite species was procured. Many key informants attributed the decrease in cases beginning in 2006 to more accurate malaria diagnosis.

Implementation of RDTs, however, faced some training challenges. In all three regions, key informants reported that some health workers were still using clinical diagnosis, and felt that RDT procedures took too much time. When the new type of RDT was procured in 2011, trainings for health workers were delayed and some nurses continued to follow directions for the previous brand. Overall it was felt that there was a lack of oversight for proper use of diagnostic procedures at health facilities. To address these issues, the NVDCP redesigned the case management training and new trainings were rolled out in the endemic regions, including new job aids such as algorithm charts and RDT quick reference guides. In addition, a mentorship program supported RDT usage by health workers [23]. As the country moves toward elimination, the NVDCP aims to achieve 100% confirmed diagnosis of all suspected cases. RDTs will also be included in the quality assurance system.

Other activities during the study period attempted to further improve case management. The Omusati program created a malaria task force to discuss cases in monthly meetings. In Ohangwena, patients waiting for care were given health education. Education was also seen as important in Omusati, where key informants called for more IEC and community outreach to increase awareness and knowledge.

Prior to 2005, chloroquine was the first line treatment for *Pf*, and sulfadoxine pyramethamine (SP), or oral quinine for pregnant women, was the second line treatment. However, increasing resistance to chloroquine led to a treatment policy change to artemisinin combination therapy (ACTs) in 2005, which was rolled out nationwide in 2006. By 2009, 94% of all health facilities in Namibia offered malaria treatment with ACTs.

Stockouts of commodities seem to be limited. In 2009, only 2% of all health facilities reported having stockouts of ACTs [24]. Only in Ohangwena did key informants report stockouts of SP and RDTs, which they attributed to a lack of inventory monitoring and proper forecasting. Facilities alleviated stockouts by requesting commodities from nearby hospital pharmacies. In all three regions, diagnosis and treatment costs declined from over half of total malaria expenditures to 24.8% in Kunene, 23.6% in Ohangwena, and 34.0% in Omusati. The decline is likely due to increased laboratory case confirmation, and reduced treatment of non-malaria febrile illness, thus procurement and expenditures for malaria treatment went down. However, challenges still exist: for example, in Omusati, healthcare providers reported that malaria patients tended to be admitted at later stages of illness, especially those patients traveling from Angola, and required more intensive care.

### Surveillance/reporting

The NVDCP has relied upon passive case detection in the public sector to identify new malaria infections. Expenditures on surveillance activities were similar in Ohangwena and Omusati, remaining relatively steady from about 4-5% from 2009 to 2011. The percentage of program expenditures for surveillance in Kunene increased from 6.8% in 2009 to 11.5% in 2011, suggesting an initial program restructuring toward malaria elimination.

Namibia’s nationwide Health Information System (HIS) collects data on inpatient and outpatient cases and deaths from regional and district public facilities, relying on data entered by a designated HIS officer at each level of government. Because reporting was often infrequent, delayed, and lacked adequate case information, the NVDCP introduced a parallel weekly surveillance system in 2010 in which district DSP focal persons compiled surveillance forms with additional key indicators (e.g. number of fevers tested, patient age, local or non-local case origination). However, the DSP focal person is also responsible for reporting on HIV/AIDS and tuberculosis, which, according to key informants, requires a disproportionate amount of time. Moreover, even though these data flow from districts to regional and national levels, they are not analyzed and information that could facilitate intervention targeting does not flow back down to district programs. Vector control data is also kept separate from case data, preventing comprehensive analysis of all program activities.

Across all regions, spending on M/M&E declined between 2009 and 2011. The percentage of spending in Kunene dropped from 13.9% to 10.4%, respectively, while that in Ohangwena (13.4% to 8.7% respectively) and Omusati (11.6% to 6.7% respectively) decreased by a slightly larger degree. Key informants cited insufficient personnel and time for completing M&E activities, relegating record keeping to a lower priority and resulting in incomplete reporting of patient register data. Management and supervision activities were also constrained; quarterly supervisory visits by regional officials to health facilities usually only occurred once a year.

### Cross border

Higher malaria caseloads in the regions adjacent to Angola are partially attributable to the fluid movement of people across the border. Angolans are believed to cross into Namibia to access healthcare because of poorly equipped and staffed facilities in Angola, resulting from the long running civil war. Crossing the border is easy and legal—a border resident card grants access to areas within 60km of the border without a passport to residents along the border in both countries [25]. While Ohangwena and Omusati have official border crossing posts, the border is porous and can be crossed at any point.

According to key informants, most malaria cases in the three study regions are believed to originate from Angola, but official statistics do not exist for the study period. Angolan patients may provide incorrect contact information, possibly to pay a lower hospital admission fee, which makes case follow up and active case detection not feasible although still very important. In addition, many Angolan villages have the same names as Namibian villages, so nurses may incorrectly assume that patients live in Namibia. Thus, key informants reported the need to synchronize malaria program activities with their Angolan counterparts. However, key informants in all regions reported communication difficulties due to language barriers and a lack of awareness of the Angolan guidelines for malaria case confirmation and management.

The Trans-Kunene Malaria Initiative (TKMI) aims to address these issues and increase coordination between the Namibian and Angolan malaria programs. TKMI is a collaboration between the governments of Namibia and Angola that aims to reduce malaria cases in five border regions: Ohangwena, Omusati and Kunene in Namibia; and Cunene and Namibe in Angola. In Namibia, TKMI would facilitate national elimination by helping to reduce malaria importation. In Angola, TKMI would help to strengthen malaria control in the south of the country, laying the groundwork for increased control of malaria in the north where transmission is even higher.

The Namibian and Angolan Ministers of Health jointly developed a concept paper in 2009 and signed a Memorandum of Understanding on April 25, 2011 [26]. The first TKMI stakeholder meeting took place in April 2011, which established the national coordinating structures in both countries, and the first joint activities – LLIN distribution and synchronized IRS – took place later that year.

Comprised of representatives from both country’s malaria programs (at district and regional/provincial levels), NGOs, immigration or military divisions, and regional technical advisory bodies, the Management and Coordination Committee is responsible for providing oversight, accountability and coordination. Trade and law enforcement bodies are responsible for issuing TKMI identity cards that help vehicles move quickly through border posts. This committee also directs the operations and the development of the Technical Committee, which is responsible for ground operations and the development of operational and research plans, including behavior change communication campaigns, surveillance/monitoring and evaluation, data management and reporting, and GIS and mapping. In addition, the Technical Committee is tasked with developing proposals for resource mobilization and work tools, such as strategic frameworks, guidelines, policies, assessments, and surveys.

On August 14, 2012 Angolan and Namibian Ministers of Health met and signed the Ondjiva Declaration on the Trans-Kunene Malaria Initiative during the second annual stakeholder meetings [27], which emphasized the need for resource mobilization and formation of partnerships at regional, provincial and district levels in order to accelerate universal coverage along the common border through IRS, LLIN distribution, case management, and social mobilization.

Although TKMI was formalized in 2009, implementation did not occur until 2011. TKMI activities had occurred only in Ohangwena until expansion into Omusati in 2013, and have primarily focused on LLIN distribution carried out by an NGO partner; distribution has been slower on the Angolan side. In addition, IRS workers have traveled to Angola to observe their vector control activities, and Angolan workers have participated in IRS trainings in Ohangwena. In Kunene and Omusati, activities have not yet been synchronized with Angola and many key informants were not aware of the existence of TKMI.

Monitoring of cross-border activities—the responsibility of the regional program, with little to no involvement of district programs—has been hampered by a lack of resources and personnel. One position for an Environmental Health Assistant at the Oshikango border crossing in Ohangwena was only filled in 2013; similar positions in Omusati have yet to be filled. There is currently no such dedicated position in Kunene.

## Discussion

From 2001 to 2011, total reported malaria cases in Namibia declined by 97.4% and API declined from 421.6 to 10.8. NVDCP key informants have attributed some of this reduction to the introduction of RDTs for more accurate malaria diagnosis and reporting. In the three study regions—Kunene, Ohangwena and Omusati—declines in malaria program spending from 2009 to 2011 mirrored similar decreases in regional APIs over the same time period. The sharpest decline in API (96.5%) and spending (53.3%) occurred in Ohangwena; the smallest decreases in API (56.7%) and spending (24.4%) were observed for Omusati.

IRS and LLIN distribution remain the primary vector control strategies of the NVDCP and accounted for a large and increasing proportion of malaria program expenditures. By 2011, vector control and prevention accounted for 45% to 58% of total malaria program expenditures in the three study regions. Total population coverage of IRS was fairly low, but the programme covered the majority of the target population. LLIN coverage averaged 32% across the study regions in 2011. Key informants cited a variety of operational constraints, including the misunderstanding, misuse, or refusal of LLINs, and for IRS, lack of training, shortages of personnel, logistical difficulties during the rainy season, and low community acceptability. To improve IRS implementation, the NVDCP plans to introduce Geographic Information Systems (GIS) software that enable better tracking of structures sprayed [18]. Because of the primary vector’s tendency to feed and rest both indoors and out, the effectiveness of IRS and LLINs must be closely monitored. Insecticide susceptibility tests carried out in 2002–2004 indicated that *An. arabiensis* is still highly sensitive to both DDT and deltamethrin (resulting in 98-100% mortality) [28]. However, alternative vector control methods such as personal protective gear or cattle spraying may need to be explored [29].

While Namibia has a national goal for elimination by 2020, the relatively low spending on surveillance activities suggests that the transition of the program from control to elimination is still in the early stages: by 2011, spending on surveillance was 4% to 12% of total expenditures across study regions. Passive case detection in the public sector is the primary method, and active case detection is in the planning stages [18]. Experiences in other countries (e.g. Sri Lanka, the Philippines) suggest that the proportion of expenditures on surveillance will increase while other costs, such as vector control, will decline, as malaria elimination progresses [30, 31]. In Namibia, major surveillance challenges remain, including reporting delays and inconsistent case investigation practices. To achieve zero transmission, case origins should be determined through comprehensive investigations followed by reactive case detection to find other infections, including asymptomatic infections that would not otherwise be identified [3, 32]. These surveillance methods are needed to better target clusters of infection and high-risk populations. The GFATM Rolling Continuation Channel (RCC) Phase II Grant in Namibia is allocated mostly to surveillance service delivery, comprising 49% of the new grant [33]. New surveillance guidelines were drafted at the end of 2013 that seek to address these gaps in the program.

To date, the NVDCP has not clearly defined the groups targeted for malaria control activities. For example, for IRS the current goal is to achieve 95% coverage in the moderate endemic regions and 100% in identified foci in the low transmission regions [18], without further guidelines for at-risk populations. Given numerous operational constraints documented and the relatively low coverage of vector control interventions, the program may benefit from evidence-based targeting of at-risk populations, leading to more efficient use of resources [31, 34]. In the three study regions, mobile populations along the northern border zone and pastoral populations who do not benefit from standard IRS or LLINs have not been effectively targeted for malaria surveillance and case management. New technical solutions may be helpful, including LLINs better suited for mobile individuals, an example of which is the usage of long-lasting insecticide treated hammocks in the forests of Cambodia [35]. Improved screening methodologies, such as network-based sampling, could be more effective and efficient in identifying infections in mobile populations [36]. Additional community engagement could help to foster acceptability of vector control measures and willingness to participate in malaria screening.

Across the study regions, references to improved human resources management were common, particularly with respect to staffing shortages, inadequate training, and more regular supervision. The percentage of spending on personnel decreased across from an average of 68% in 2009 to 59% in 2011. In contrast, expenditure studies in other eliminating countries show a trend toward a greater proportion of spending on personnel during the CLM phase [30, 31], through the elimination phase, and into prevention of reintroduction after elimination is achieved [31]. Additional capacity building may improve the quality of diagnosis and treatment and IRS. The program is currently adding new team members for surveillance, clinical malaria, and vector control. In addition to needs for greater human resources, greater communication and coordination across program levels and partners is needed; many regional- and district-level key informants were not aware of TKMI, the major cross-border initiative with Angola.

Namibia lies between diverse malaria transmission zones—Angola to the north is considered endemic while South Africa to the south and Botswana to the east have very low transmission. Of a number of southern African regional malaria initiatives designed to address cross-border transmission, only one, the Lubombo Spatial Development Initiative (LSDI, involving Swaziland, South Africa, and Mozambique) has reported some successes [37]. Namibia is currently involved in three regional initiatives: the TKMI, the Trans-Zambezi Malaria Initiative (TZMI, involving Angola, Botswana, Namibia, Zambia, and Zimbabwe) [38] and the Elimination Eight (E8, involving the eliminating countries of Botswana, Namibia, South Africa, and Swaziland, and their northern neighbors Angola, Mozambique, Zambia, and Zimbabwe) [39]. Despite securing commitments from all participating countries, TZMI and E8 have not yet coordinated any border-focused activities, and coordinated activities for TKMI have only just recently begun in 2011. Given the high level of political commitment to these regional initiatives, it is hopeful that they will contribute to the reduction of malaria importation into Namibia and help the NVDCP to reach malaria elimination.

### Limitations

The results of this case study should be interpreted in light of several caveats. Results are based on a small, select sample of regions and cannot be generalized to reflect the program strategies, activities, or expenditures for other regions or for the country as a whole. When there was a NVDCP representative present during interviews, key informants may have responded to questions differently than if unsupervised. Costs incurred by partner organizations or private sector health facilities were not included in the expenditure data, nor were household expenditures on malaria.

## Conclusions

As Namibia moves toward malaria elimination, there are many operational constraints that must be addressed. In addition to allocating sufficient human resources to vector control activities, developing a greater emphasis on surveillance is central to the ongoing program shift from control to elimination, particularly in light of the malaria importation challenges experienced in the northern border regions. Steps toward building more robust surveillance is already underway, enabled by additional GFATM funding and matching domestic financing resources [22]. Building skills and processes for case management and its supervision was a priority in 2012 and 2013. The NVDCP plans to increase the number and capacity of surveillance officers and clinical mentors in malarious regions, develop surveillance guidelines to standardize case investigation, active case detection, and reporting indicators, and improve the M&E structure by linking the different data capture systems and conducting data analysis [18]. While overall program resources may continue on a downward trajectory, the program will be well positioned to actively eliminate the remaining foci of malaria if greater resources are allocated toward surveillance efforts.

## Appendix A

### Map of P. falciparum transmission and predictions of receptive PfPR_2–10_

Three previously described criteria were used to define the limits of stable malaria transmission in Namibia [40]. These were: the suitability of ambient temperature; aridity; and medical intelligence. The resulting map classified areas in Namibia into those that are unsuitable for transmission (dark grey), those that support unstable transmission (light grey) and areas of stable transmission (the rest of the country).

In 2011, village-level data on mass blood examinations undertaken between 1967–1992 were assembled from monthly and annual reports of the parasitology department at the National Institute of Tropical Diseases (NITD) at Tzaneen, South Africa. Information on village name, month and year of the survey, number of people examined, number positive for *P. falciparum*, and the age range of the surveyed community were extracted. The longitude and latitude of all survey locations were subsequently identified using a variety of digital place name databases, gazetteers, and a settlement database mapped using Global Positioning Systems (GPS) receivers. Model-based Bayesian geostatistical methods were used to map continuous surfaces of malaria risk at 5 × 5 km spatial resolution for the years 1969, 1974, 1979, 1984 and 1989 within the limits of stable transmission [9]. These were then combined to generate a single map of maximum mean *Pf*PR_2–10_ at each grid location. The mean maximum *Pf*PR_2–10_ was computed for each health district and used to classify these geographic units by *Pf*PR_2–10_ receptive risks [13].

### Research team and reflexivity

The following authors (along with their credentials and positions at the time the research was undertaken) conducted the key informant interviews:

- CL (MHP) is a malaria program health specialist that supports the NVDCP;
- MG (BA) is a research assistant with past experience working with Namibia’s Ministry of Health and Social Service and NVDCP

CL underwent a four-day training prior to the commencement of research activities. All interview guides, data tracking forms, and data processing procedures were pre-tested in the Oshikoto region before being administered in the study regions. CL subsequently trained MG in study and interview protocols when data collection activities were launched.

With assistance from NVDCP national-level management, interviewers were introduced to potential key informants at regional- and district-level offices in the selected provinces. The NVDCP also provided letters of introduction authorizing the research to take place and to facilitate introductions to local program offices. Researchers introduced themselves and explained the objectives of the study to each potential study participant. For key informants who provided verbal informed consent to participate in the study, interviewers noted their current and former position in relation to the malaria program.

### Study design

The design of the case study was based on a grounded theory approach to elicit success factors and challenges that the malaria control program has encountered in its transition from malaria control to elimination. In this effort, financial resources were identified as one key dimension of understanding the constraints (or lack thereof) under which program choices were made. At each office or site visit, additional data regarding program expenditures, epidemiological indicators, or intervention coverage were collected for the selected sample years. Each interview was conducted by at least two researchers; all were conducted in English and audio-recorded. Interviews lasted from 30 minutes to three hours, depending on the participant’s degree of knowledge and experience. Written notes taken during the interview were then combined with audio recordings for later data analysis.

A coding scheme was developed to categorize interview content into themes, which were pre-defined based on past research experience in conducting other case studies in this series. After discussions with GN and JL, interview content was reviewed and categorized by MG.

### Expenditure calculations

Personnel expenditures reflect salary amounts for each employee; information on benefits was not comprehensively available. Percent time spent on malaria activities was estimated based on the estimated malaria burden in each district from 2009 to 2011 (i.e. the proportion of reported malaria cases among reported febrile patients) per standard operating procedures of the NVDCP^a^ combined with first-hand knowledge of the job responsibilities for each employee (e.g. medical director, nurse, spray man). Time allocations across activity types (e.g. M&E, surveillance) were estimated based on a combination of Ministry of Health and Social Services national policy guidelines, terms of references, and key informant responses.

Expenditures for consumables used in diagnosis and treatment were calculated based on a standard formula of supplies required to perform one blood smear at prevailing purchase prices and the number of blood smears conducted. Drug quantities were obtained from the Central Medical Store and regional pharmacists. Omusati drug quantities were incorporated within Oshana region’s drug expenditures, so a ratio of Omusati malaria cases to Oshana cases was applied to calculate Omusati’s costs for RDTs, artemether lumefantrine, quinine, and sulfadoxine pyrimethamine. Insecticides and other equipment used during vector control activities were obtained from the NVDCP and regional environmental health officers. LLINs and spray equipment were assumed to have a greater than one year useful life; thus, straight line depreciation was utilized with a 3% discount rate and a three year and five year useful life, respectively. When calculating coverage of LLINs, each net was assumed to cover two persons for three years.

Expenditures for health office utilities and maintenance were collected at regional administrative offices. For months where no receipt of expenditure could be found, either on its own or within another month’s bill, an average was calculated and added to the ^a^ Fever is an indicator that is recorded in each health facility registry and is used as a proxy for total healthcare burden per facility, per health district. This proportion is used to measure performance regionally, nationally, and for external evaluation with donors like the Global Fund. yearly amount. The estimated commercial value of real estate was not captured, as reliable estimates could not be obtained from key informants and records were not available at health offices. Values of capital equipment for furniture, computers, and microscopes were not available in all regions, but estimates of vehicles used by program activities were estimated based on useful life years remaining and current resale value. For each region, a vehicle master list was obtained that included year, make, and model, as well as the region’s main purpose for each vehicle. Assuming the year of the vehicle to be the purchased year, current value was depreciated to find a base year cost. From there, straight line depreciation using a 3% discount rate and useful life of ten years was applied to find the depreciated yearly value for each sample year. To determine the number of hours the vehicle was used specifically for malaria control, the average personnel time spent on malaria was used as an estimate of percent time spent on malaria, and activity allocation was determined by the vehicle’s main purpose.

## Abbreviations

ACT: Artemisinin combination therapy
API: Annual parasite index
CLM: Controlled low-endemic malaria
DDT: Dichloro-diphenyl-trichloroethane
DSP: Directorate of Special Programmes
E8: Elimination 8
EHO: Environmental Health Officer
GFATM: Global Fund to Fight AIDS, Tuberculosis and Malaria
GIS: Geographic Information Systems
HIS: Health information system
HIV/AIDS: Human immunodeficiency virus/Acquired immunodeficiency syndrome
IEC: Information, education, and communication
IRS: Indoor residual spraying
ITN: Insecticide-treated net
LLIN: Long-lasting insecticide-treated net
M/M&E: Management/Monitoring and Evaluation
MoHSS: Ministry of Health and Social Services
NGO: Non-governmental organization
NIP: National Institute of Pathology
NVDCP: National Vector-borne Diseases Control Programme
PAR: Population at risk
*Pf*: *Plasmodium falciparum*
*Pf*PR_2–10_: *Plasmodium falciparum* parasite rate (for age range 2–10 years)
PPY: Per population at risk per year
RDT: Rapid diagnostic test
SP: Sulfadoxine pyramethamine
TKMI: Trans-Kunene Malaria Initiative
TZMI: Trans-Zambezi Malaria Initiative.
